# *Hypericum perforatum*: Synthesis of Active Principles during Flowering and Fruitification—Novel Aspects of Biological Potential

**DOI:** 10.1155/2017/2865610

**Published:** 2017-11-22

**Authors:** Nebojša Kladar, Jasminka Mrđanović, Goran Anačkov, Slavica Šolajić, Neda Gavarić, Branislava Srđenović, Biljana Božin

**Affiliations:** ^1^University of Novi Sad, Faculty of Medicine, Department of Pharmacy, Hajduk Veljkova 3, 21000 Novi Sad, Serbia; ^2^University of Novi Sad, Faculty of Medicine, Oncology Institute of Vojvodina, Put Dr Goldmana 4, 21203 Sremska Kamenica, Serbia; ^3^University of Novi Sad, Faculty of Sciences, Department of Biology and Ecology, Trg Dositeja Obradovića 3, 21000 Novi Sad, Serbia

## Abstract

St. John's wort is a widely used medicinal plant. The quality of herbal drug, which is in most of the cases collected from nature, varies. Therefore, the aim of the present study was detailed chemical characterization of* Hypericum perforatum* subsp.* perforatum* samples collected in close time intervals during flowering and fruitification with the purpose to state the phenological stage characterized by maximum levels of active principles. The antioxidant potential and potential to inhibit biologically important enzymes, as well as the cytotoxicity and genotoxicity of the sample collected during the full flowering period, were evaluated. Data showed that the optimal period for the achieving of maximum level of active principles is the phenophase between floral budding and flowering stage. Significant antioxidant potential and the ability to inhibit biologically important enzymes (especially *α*-glucosidase) were recorded. The extract exhibited no genotoxicity in subcytotoxic concentrations, while increased cytotoxicity recorded in cotreatment with bleomycin on malignant cell lines was especially significant.

## 1. Introduction

The genus* Hypericum* includes more than 500 species, classified into 36 sections [1]. The best known representative of the genus is St. John's wort (*Hypericum perforatum* L. 1753, Hypericaceae), a species widely used in traditional and conventional medicine. To date conducted* in vitro* and* in vivo,* as well as clinical, studies suggest antioxidant, antiviral, antifungal, antibacterial, wound-healing, antidepressant, and many more properties of* Hyperici herba* [2]. Two basic forms of preparations based on* H. perforatum* are being used. Oil macerates are, when applied externally, intended for treatment of different skin changes, while internal application is recommended in the case of stomach and bile disorders, inflammation of respiratory and urogenital system, migraine, diabetes, and so forth. However, of particular importance are water and water-alcoholic extracts which exhibit clinically proved antidepressant activity [3, 4]. Considering the overall multitargeting therapeutic potential, a constant raise of demand for* Hyperici herba* is present on the world market. The most important secondary metabolites present in the drug are phloroglucinols (hyperforin, adhyperforin), naphtodianthrones (hypericin, pseudohypericin), flavonoids (rutin, quercetin, quercitrin, isoquercitrin, hyperoside, and amentoflavone), phenolic acids, and small amounts of essential oil. The content of active principles in plants varies depending on different ecological factors characteristic for plant habitat, as well as plant development. Usually,* H. perforatum* is not being cultivated but rather collected from nature which leads to differences of herbal drugs from the aspect of chemical quality. The recommendation is that the collecting of plant material should be performed at open habitats during the period of flowering. However, long postfloral retention of corolla and differences in types of vegetation induce the existence of different stages of flowering at the same time point, which leaves a space for variations of levels of active principles in herbal drug [5]. As known to the authors, no studies which provide detailed description of active principles variation in* Hyperici herba*, related to the phenological development of biological source, were conducted. Usually, only the differences in chemical profiles of the plants collected before, during, and after flowering are being reported [6].

Considering the overall safety of* Hyperici herba*, the results of* in vitro* tests suggesting new potential medical indications of* H. perforatum* are of high importance. Previous reports state the inhibitory activity of water-alcoholic extracts of different* Hypericum* species on acetylcholinesterase (AChE), suggesting the potential beneficial effects in patients suffering from Alzheimer's disease (AD) [5, 7]. Since AD is commonly associated with depression, preparations based on* Hyperici herba* could have significant therapeutic values. Also, the raise of incidence of* diabetes mellitus* (DM) on the world scale [8] points a great attention toward new drugs which can affect the metabolism of sugars. There are reports of* Hypericum* extracts inhibiting *α*-glucosidase and *α*-amylase which could lead to decrease in glucose absorption and consequently to lowering blood sugar levels [5].

Cancer represents one of the most frequent causes of mortality around the world, with the prognosis of increase to 70% until 2030. [9]. This combined with, so far unsatisfying effects of available chemotherapy and radiation therapy, leads toward constant search for new anticancer drugs, especially of natural origin [10]. The attention of researchers is directed to antiproliferative effects of herbal preparations used in traditional medicine, as well as to the effects of combined administration of compounds of natural origin and antineoplastic drugs. Possible benefits of such therapy could be reflected through lowering the dosage of conventional medicines and consequently lowering the toxicity of antineoplastic drugs on healthy cells followed by higher cytotoxic effects toward cancer cells. It is well known that biologically active compounds originating from plants can promote the gene expression which would result in multiplication of conventionally used medicines [11].

The aim of the present study was detailed chemical evaluation of secondary metabolites accumulation during flowering and fruitification of St. John's wort. The biological potential of the water-alcoholic extract obtained from* H. perforatum* subsp.* perforatum* collected at full flowering stage was assessed through antioxidative potential and potential to inhibit *α*-amylase, *α*-glucosidase, monoamine oxidases A and B (MAO-A and MAO-B, resp.), and AChE. Also, cytotoxic potential of the extract and combination of the extract and antineoplastic drug (bleomycin) on malignant cell lines was evaluated, since no reports describing this cotreatment were found. For the evaluation of potential DNA damage and overall safety, genotoxic potential of the evaluated extract on peripheral blood lymphocytes was estimated.

## 2. Material and Methods

### 2.1. Plant Material and Obtaining of the Extracts

Plant material included 22 specimens of* Hypericum perforatum* subsp.* perforatum* grown in wild near Padej, Republic of Serbia (45°50′59.53′′N, 20°9′53.51′′E). The vouchers are identified and deposited in BUNS (Herbarium of the Department of Biology and Ecology, Faculty of Natural Sciences and Mathematics, University of Novi Sad). In close time intervals, starting from 20 April until the 6 August 2014, aerial parts of the plants were collected. Water-alcoholic extracts of 22 plant samples (Table 1) are obtained by maceration with 70% ethanol for 72 h. The residues of solvent are evaporated and dry extracts (d. e.) are dissolved in absolute methanol prior to chemical characterization by liquid chromatography. Sample 12, collected at the stage of full flowering, was extracted as previously described, evaporated to dryness and d. e. was dissolved in water (HP) for further evaluation of biological potential, cytotoxicity and genotoxicity, and estimation of levels of total phenolics and total flavonoids.

### 2.2. Chemical Characterization of the Extracts

#### 2.2.1. Total Phenolics and Flavonoids

The amounts of total phenolics and flavonoids in the HP are quantified as previously described [5] and expressed as mg of gallic acid equivalents (GAE) per g of dry extract (d. e.) and mg of quercetin equivalents (QE) per g of d. e., respectively.

#### 2.2.2. HPLC Analysis

**Figure 1:**
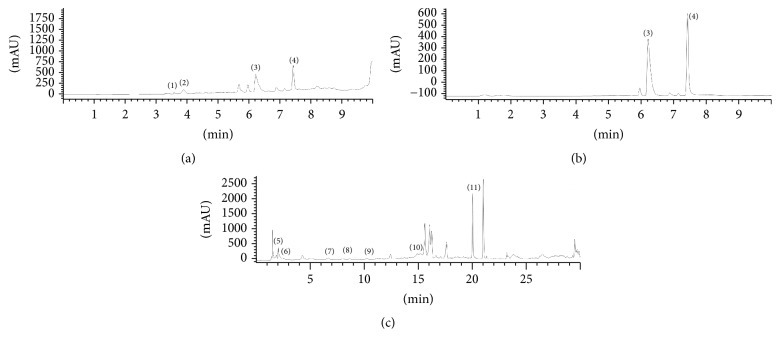
HPLC-DAD chromatograms of sample 12 obtained by method I: (a) detection at 270 nm and (b) detection at 590 nm and method II: (c) detection at 280 nm. Detected compounds: (1) NA, (2) Am, (3) Hpf, (4) Hpc, (5) R, (6) GA, (7) CHA, (8) PHB, (9) CA, (10) FA, and (11) Qe.

Two methods of liquid chromatography coupled with diode array detector (HPLC-DAD) are used for qualitative and quantitative profiling of plant extracts (Figure 1). The experiments are performed on Agilent HP 1100 HPLC-diode array detection (DAD) system equipped with an autosampler (Agilent, Waldbronn, Germany), while the components of interest were separated using reversed-phase Zorbax CB-C18 column (4.6 × 150 mm, i. d., 5 *μ*m particle size) held at 25°C. Method I, previously described by Božin et al. [3], was used for quantification of hypericin (Hpc), hyperforin (Hpf), apigenin (Ap), amentoflavone (Am), and naringenin (NA). Method II was developed based on a report by Ziaková and Brandšteterová [12] and used for determination of quercetin (Qe), rutin (R), epicatechin (Ec), caffeic (CA), chlorogenic (CHA), ferulic (FA), gallic (GA), and* p*-hydroxybenzoic acid (PHB). Gradient elution was applied (3.25 min, 0% B; 8 min, 12% B, 15 min, 25% B, 15.8 min, 30% B, 25 min, 90% B, and 25.4 min, 100% B) with the flow rate of 1 mL/min, where solvent A was 0.1% solution of acetic acid in water and solvent B was 0.1% solution of acetic acid in acetonitrile. Before the injection of extracts, calibration curves of all chemical standards of quantified compounds were obtained.

### 2.3. Antioxidant Potential

#### 2.3.1. Radical Scavenging Capacity (RSC)

The ability of HP to neutralize 2,2-dipheny-l-picrylhydrazil (DPPH), hydroxyl (OH), and nitroso (NO) radicals is estimated spectrophotometrically as previously described [5]. Briefly, different concentrations of the extract are added to the DPPH^*∙*^ solution and the disappearance of purple color was followed at 515 nm. In the OH-test, free radicals are formed in Fenton's reaction and degradation of 2-deoxy-D-ribose followed by formation of malonyl-dialdehyde (MDA) was evaluated spectrophotometrically at 532 nm. Nitroso radicals are generated after addition of sodium nitroprusside in the reaction mixture, while the antioxidant potential was estimated upon addition of Griess's reagent which forms purple complex with NO^*∙*^.

#### 2.3.2. Inhibition of Lipid Peroxidation (LP)

The potential of HP to inhibit the process of lipid peroxidation is estimated as in previously published research by Kladar et al. [5]. Liposomes are used as a test-model of biological membranes while OH^•^ are formed in Fenton's reaction.

The obtained results for the HP were compared with ascorbic acid and synthetic antioxidants such as butylated hydroxytoluene (BHT), propyl gallate (PG), and quercetin dihydrate (QDH).

#### 2.3.3. Ferric Reduction Antioxidant Potential

The ability of HP to reduce Fe^3+^ to Fe^2+^ is estimated based on a method by Lesjak et al. [13] in which Fe^2+^ with 2,4,6-tripyridyl-S-triazine (TPTZ) forms a colored complex with the absorbance maximum at wavelength 593 nm. The same procedure was used for determination of ferric reduction potential of ascorbic acid since the antioxidant potential of the extract was quantified as the milligrams of the ascorbic acid equivalent per gram of d. e. (mg AAE/g d. e.). All measurements are performed in triplicate.

### 2.4. Inhibition of Biologically Important Enzymes

#### 2.4.1. Inhibition of Monoamine Oxidase A and Monoamine Oxidase B

The inhibitory potential of the HP against human recombinant MAO-A and MAO-B is estimated similarly as in the study by Samoylenko et al. [14]. Deamination of kynurenine, which is used as a substrate, and consequent formation of 4-hydroxyquinoline are followed spectrofluorimetrically (Ex = 320 nm, Em = 380 nm). In the MAO-A inhibition assay, the final concentrations of the enzyme and substrate in the reaction mixture were 5 *μ*g/mL and 80 *μ*M, respectively, while, in the case of MAO-B inhibition, the concentrations of enzyme and substrate were 10 *μ*g/mL and 50 *μ*M, respectively. The water solution of the examined extract was added in a single concentration to the reaction mixtures to estimate the percentage of the enzyme inhibition compared to the control which contained only the enzyme and substrate. Standards of moclobemide and selegiline are used as positive controls for MAO-A and MAO-B inhibition, respectively. All measurements are performed in triplicate.

#### 2.4.2. Inhibition of Acetylcholinesterase

Anticholinesterase potential of the HP is estimated by modified Ellman's method [3]. Final activity of the enzyme in the reaction mixture was 8.15 U/L. Two concentrations of the examined extract are added in the reaction mixture. The percentage of inhibition was calculated compared to the reaction mixture which contained no extract. Galantamine is used as a positive control. All measurements are performed in triplicate.

#### 2.4.3. Inhibition of *α*-Amylase

Inhibition of *α*-amylase activity by the HP is evaluated as previously described [5]. Reaction mixtures contained Starch azure® (substrate), porcine *α*-amylase (final reaction mixture activity 0.6 U/mL), and sodium phosphate buffer (pH = 7.2) with NaCl. Two concentrations of the extract are added in test tubes. The percentage of inhibition is calculated compared to the absorbance of reaction mixtures containing no extract. Acarbose is used as a positive control. All the measurements are done in triplicate.

#### 2.4.4. Inhibition of *α*-Glucosidase

The potential of the examined extract to inhibit *α*-glucosidase is estimated by the modified method of Sigma-Aldrich [15]. The assay mixture contained potassium phosphate buffer (pH = 6.8), glutathione (reduced solution), and *α*-glucosidase from* Saccharomyces cerevisiae* obtained from Sigma-Aldrich and* p*-nitrophenyl-*α*-D-glucoside (PNP-Gluc), which was used as a substrate. Two different concentrations of the HP are added in the reaction mixture. After the incubation of 20 min at 37°C the reaction was stopped by addition of Na_2_CO_3_ and the absorbance of solution was spectrophotometrically measured at 400 nm. Reaction mixture without the examined extract was considered as a 100% enzyme activity. Final activity of the enzyme in the reaction mixture was 7.6 U/L. Acarbose was used as a positive control. All the measurements are performed in triplicate.

### 2.5. Cytotoxicity and Genotoxicity

#### 2.5.1. Growth and Culture of Cells

The estimation of cell growth activity is performed on one untransformed human cell line MRC-5 (fetal lung fibroblast, ECACC 84101801) and three human malignant transformed cell lines: HeLa (cervix epithelioid carcinoma, ECACC number 93021013), HT-29 (colon adenocarcinoma, ECACC number 91072201), and Hs-294T (melanoma, ATCC HTB-140). The cell lines were grown and maintained in DMEM (Sigma-Aldrich, USA) medium supplemented with FCS (10%), penicillin (100 units/mL), and streptomycin (100 *μ*g/mL), being referred to as complete medium. Furthermore, the cells were cultured in 25 cm^2^ flasks at 37°C in the atmosphere of 5% CO_2_ and high humidity and subcultured twice a week. A single cell suspension was obtained using 0.1% trypsin with 0.04% EDTA.

Heparinized whole blood, collected by venous puncture from 40-year-old healthy female donor not having been exposed to any chemical or physical agent during the last 6 months, is used for the analysis of genotoxic potential. Briefly, 0.5 mL of the whole blood was added to 5 mL of the Lymphochrome, lymphocytes culture medium (Lonza, BioWhittaker™), supplemented with phytohemagglutinin. Lymphocyte cell culture of peripheral blood was incubated at 37°C for 72 h in 5% CO_2_ atmosphere with 95% humidity.

#### 2.5.2. Cytotoxicity Assay

Cytotoxicity of HP is evaluated by the colorimetric sulforhodamine B (SRB) assay as previously described [16]. Cell lines are plated into 96-well microtiter plates at different seeding density of 5 × 10^3^ cells per well for MRC-5 and Hs-294T, 4 × 10^3^ cells for HeLa, and 6 × 10^3^ cells for HT-29 in a volume of 180 *μ*L and preincubated in complete medium supplemented with 5% FCS, at 37°C for 24 h. For the evaluation of the cell growth activity, the extract was diluted in NaCl to obtain final concentrations in range of 0.1–600 *μ*g/mL, while the control group contained only NaCl. All cell lines were previously treated with bleomycin (BLM) in order to determine EC_50_ values. During the examination of combined treatment (HP and BLM) effects on cell cultures, the final concentration of BLM was 100 *μ*g/mL. Serial dilutions of HP and BLM (20 *μ*L/well) were added to achieve required final concentrations. Microplates are then incubated at 37°C for an additional 48 h. The development of color was spectrophotometrically measured at 540 nm against 620 nm as background. The results of cell growth activity were expressed as mean ± SD of experiments performed in octuplicate. The effect on cell growth was expressed as a percent of the control and calculated as

(1) $$\begin{array}{c} \left(\;\frac{At}{Ac}\;\right)\times 100\left(\;\%\;\right), \end{array}$$

where At is the absorbance of the test sample and Ac is the absorbance of the control. Using EC_50_ values obtained in a nontumor cell line and in the respective tumor cell line, nontumor/tumor EC_50_ ratios (NT/T) were calculated for the extract, drug, and combination of the extract and drug.

#### 2.5.3. Genotoxicity

Genotoxicity of HP is evaluated by cytochalasin block micronuclei assay (CBMN) which was performed according to published procedures [17] with minor modifications. The peripheral blood lymphocytes culture was set up in triplicate vessels per concentration for each experimental treatment condition. According to the recommendations for testing the genotoxic effects of compounds [18], the extract was added after 20 h of preincubation. The final concentrations of the extract in peripheral blood culture were 50, 100, and 200 *μ*g/mL. After 44 h of culture setup, the medium was replaced with fresh one containing cytochalasin-B (CytB) at a final concentration of 6 *μ*g/mL and incubation continued for the next 24 h. Thereafter, cells were exposed to a cold hypotonic solution (0.56% KCl) and fixed three times with methanol : glacial acetic acid (3 : 1, v/v). Air-dried slides were stained with 2% Giemsa in distilled water for 9 min. The CBMN assay was performed, analyzing more than 1000 cells for each sample. Standard criteria were used for the identification of micronuclei (MN) [19]. Monitored values included number of mononucleated, binucleated, and multinucleated cells, incidence of micronuclei, and nuclear division index (NDI). Micronucleus incidence was presented as a number of micronuclei per 1000 examined binuclear cells.

### 2.6. Statistical Analysis

The data were presented as mean values ± SD. Concentrations of the extracts needed for the neutralization of 50% of free radicals and LP, inhibition of 50% of the enzyme activity or inhibition of 50% of cell growth (RSC_50_, IC_50_, and EC_50_, resp.) were determined by regression analysis from concentration-effect curve, using Microsoft Excel, v2010. One-way Anova and Fisher LSD test applied in genotoxicity assay, as well as principle component analysis (PCA), were performed by Statistica 13.0, StatSoft.

## 3. Results and Discussion

### 3.1. Chemical Characterization of the* H. perforatum* Subsp.* perforatum* Extracts

Preliminary chemical characterization of the HP showed that the amounts of total phenolics and flavonoids were 193.31 ± 7.85 mg GAE/g d. e. and 35.85 ± 0.84 mg QE/g d. e., respectively, which is in order with previous studies of this species [6, 7, 20]. The amounts of compounds extractable from plant samples collected during selected phenological stages varied from 10.34% to 23.57% (Table 1) and generally were decreasing during the period of plant development. Detailed chemical characterization and quantification of selected secondary metabolites by liquid chromatography (HPLC-DAD) reveal several patterns of accumulation (Table 1). Hypericin, marked as one of the main active principles, reached its maximum values in samples 12–15, which were collected during the period of flowering. These values varied from 0.70 to 0.88 mg/g of dry herb (d. h.) and were similar as in the previously conducted studies [3, 6, 21, 22]. The termination of flowering period (samples 13–17) was followed by the decrease of hypericin levels, which corresponds to previously reported results suggesting its highest accumulation in plant material with fully opened flowers [23–26]. However, we have observed different trend of hyperforin accumulation. The highest content (0.89–0.92 mg/g d. h.) was detected in samples 9–11, obtained from plant material collected at floral budding stage, and was similar to previously reported results [3, 6, 20]. This is also in accordance with the study conducted by Gioti et al. [20] and may be explained by the general instability and photosensitivity of hyperforin molecule [27]. Opposite are the results of the study conducted by Cirak et al. [25] and Çirak et al. [28] who report the maximum of hyperforin content in the period of blossom and a study conducted by Filippini et al. [6] who state the maximum level of this secondary metabolite during the period of fruit development, although without specifying the* H. perforatum* subspecies examined. However, it is notable that, in our research, the content of hyperforin decreases after opening of flowers but, three weeks after, again starts to increase, which might partially be correlated with fruiting or with the development of new flowers. The levels of rutin reach their maximum value of 0.15 mg/g d. h. at the period before flowering (sample 5) after which they decrease, as already reported by Bagdonaitė et al. [29] who state the maximum levels of rutin at the floral budding stage. The rest of the quantified flavonoids, for example, naringenin, quercetin, and amentoflavone reached the maximum levels during the floral budding or in the stage of opening of flowers. The level of apigenin decreased during the development of plants, while epicatechin is being detected only in samples collected in periods after flowering. The amounts of chlorogenic and caffeic acid reached their maximum values during the floral budding stage, while the content of gallic acid generally decreased during plant development, possibly as a result of tannins formation.

**Figure 2:**
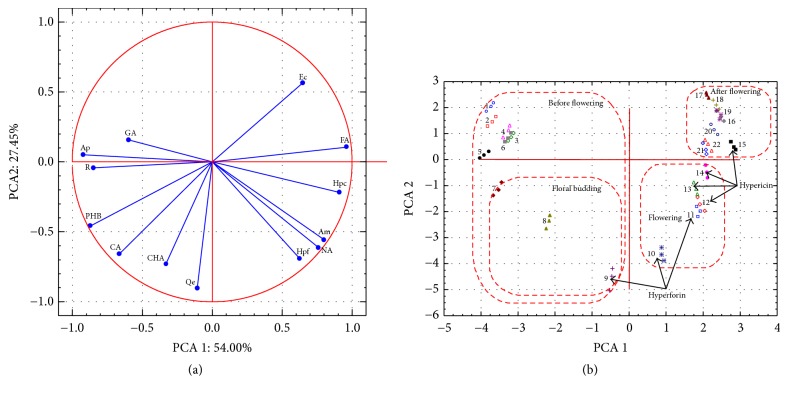
Principal component analysis. Projection of the examined variables, compounds (a) and case samples (b) in the space defined by the first and the second principal component.

Performed PCA indicates that the first component explains 54% of variance and together with the second component covers more than 80% of variance. The main compounds affecting the grouping of samples based on the load values of the first component are hypericin, apigenin, rutin, ferulic, and* p*-hydroxybenzoic acid. However, in the space defined by the second axis, the grouping is mainly a result of different quantities of hyperforin, quercetin, and chlorogenic acid in the examined samples. Particularly significant is the correlation between the detected levels of hyperforin, hypericin, amentoflavone, and naringenin which accumulate at the flowering period and are some of the main active principles of* Hyperici herba* (Figure 2(a)). The position of the analyzed samples in the space defined by the first and the second principal component clearly indicates four different phenological stages of* H. perforatum* subsp.* perforatum* (Figure 2(b)). It is obvious that the samples collected at the end of floral budding stage and the beginning of flowering stage contain the highest quantities of hyperforin followed by considerable amount of hypericin. As the plant development continues, the levels of hypericin further increase, but the accumulation of hyperforin decreases, confirming what was previously stated that the optimal period for harvesting of plant material is the transition between floral budding and flowering stage.

### 3.2. Antioxidant Potential of the Examined* H. perforatum* Water-Alcoholic Extract

Reactive oxygen species (ROS) are involved in pathogenesis of various diseases and syndromes. Generally, it is proved that plants possess significant antioxidant potential, mainly addressed to the presence of different phenolic and flavonoid compounds [5]. Furthermore, modern trends suggest the use of natural products in food and cosmetic industry as a replacement for synthetic antioxidants. However, considering the complexity of plant extracts composition and antioxidant mechanisms of action, the use of several assays for examination of ROS neutralization potential is highly recommended [3]. Therefore, in the present study, five assays were performed for the estimation of HP antioxidant potential (Table 2). In all of the applied test-systems, the examined extract managed to reach 50% of ROS neutralization and the obtained results for IC_50_ values corresponded to those previously reported [3, 7, 22]. Regarding DPPH^•^ scavenging assay, HP exhibited weaker but comparable antioxidant potential to QDH and PG. It is important to point out that DPPH^•^ is a synthetic compound, not occurring in the human body and represents a good choice only for preliminary screening of antioxidant potential [3]. Significantly lower potential of the HP, compared to synthetic antioxidants, was noticed in the neutralization of OH^*∙*^ and NO^*∙*^ and inhibition of LP, but in the FRAP-test notable antioxidant activity was reported (212.26 mg AAE/g d. e.). However, it must be emphasized that comparison of antioxidant potential in the present study was performed between pure compounds and plant extract, which is a mixture of different secondary metabolites whereby some of them do not possess potential to scavenge ROS. Considering the relevance of* in vitro* recorded antioxidant potential in concentrations up to 10 *μ*M, the examined HP represents a promising free radical scavenging agent [30].

### 3.3. Inhibition of Biologically Important Enzymes

Clinically proved antidepressant activity of* H. perforatum* water-alcoholic extracts is so far ascribed to several mechanisms which, by most of the authors, include inhibition of synaptosomal reuptake of noradrenaline, dopamine, and serotonin and inhibition of MAO-A and MAO-B [5]. Generally, scientific studies gave the advantage to the hyperforin, rather than hypericin, as the main active principle responsible for the antidepressant potential of St. John's wort-based preparations. In the present study, significantly higher potential of the examined extract to inhibit MAO-A was observed (Table 2). However, when comparing this activity with moclobemide, a synthetic MAO-A inhibitor, it may be concluded that HP is several times less potent. Since most of the patients suffering from AD also show symptoms of depression, it would be significant that* H. perforatum*, which was already proved as efficient in treatment of mild to moderate forms of depression, also shows therapeutic effects in AD. Inhibitors of acetylcholinesterase are one of the several recognized groups of medicines used in the treatment of AD. Galantamine, an alkaloid isolated from* Galanthus woronowii*, Amaryllidaceae, presents one of the most potent inhibitors of AChE. However, screening of plants and other products of natural origin which possess anticholinesterase activity is constantly in the focus. In our study, the examined extract applied in concentrations of 475 and 800 *μ*g/mL inhibited 17.22 and 42.31% of AChE activity, respectively (Table 2). These results are comparable to the results reported by Božin et al. [3] and Altun et al. [7] but significantly less promising when compared to the anticholinesterase potential of galantamine (IC_50_ = 8.56 *μ*g/mL).

Considering the widespread use of* Hyperici herba*, screening of new biological activities of this plant is more than welcome. Diabetes is one of the syndromes with rapidly rising incidence. Predictions state that the prevalence of diabetes will double in 2000–2030 period in all age groups [8]. Increased blood sugar level is the initial symptom which later progresses toward different complications. Therefore, inhibition of enzymes included in sugar metabolism is one of the established therapy approaches of diabetes. Several approved medicines utilize this pharmacological mechanism of action, but the question is whether some of the phytopreparations also possess this biological activity. The present study is one of the first reports dealing with the potential of* H. perforatum* extract to inhibit *α*-amylase and *α*-glucosidase. For both of the enzymes, at tested concentrations, IC_50_ values were reached (Table 2). However, when compared to acarbose, which was used as positive control, more significant is inhibition of *α*-glucosidase. This leaves a space for further evaluation of* H. perforatum* extracts as potent antihyperglycemic agents.

### 3.4. Cytotoxicity

The antitumor effects of HP and a combination of HP with BLM were evaluated* in vitro*, by SRB assay using human nontumor MRC-5 and three tumor cell lines: HeLa, Hs-294T, and HT-29. Considering the inhibition of tumor cells growth, moderate activity of HP was recorded (EC_50_ = 340.94–468.94 *μ*g/mL) (Table 2), which corresponds to the previously published results [31]. The ratio of selective cytotoxicity toward nontumor and tumor-cells was low (NT/T = 1.02–1.40). Furthermore, the most susceptible tumor cell line was HeLa followed by HT-29, while the lowest cytotoxic potential was exhibited on Hs-294T cell line.

The cytotoxicity of the HP could be correlated to the reported antiproliferative activity of hyperforin, hypericin, quercetin, and chlorogenic acid. Hyperforin represents a promising anticancer agent which suppresses proliferation of a number of cancer cell lines, including mammary carcinoma, squamous cell carcinoma, melanoma, glioma, fibrosarcoma, chronic myeloid leukemia, and lymphoma [32]. The hyperforin cytotoxicity is often explained by the mechanism of apoptosis [33, 34]. Schempp et al. [32] state that hyperforin induces early apoptotic events, for example, rapid loss of mitochondrial transmembrane potential and subsequent cytochrome c release which consequently leads toward reducing of size of experimental colon carcinoma metastases and inhibits proliferation in peripheral blood mononuclear cells. Molecular mechanism activated by hyperforin in chronic lymphoid leukemia and acute myeloid leukemia cells, known for their resistance to chemotherapy, is activation of the proapoptotic Bcl-2 family protein Bad and Noxa [35]. Antiproliferative effect of hyperforin* in vivo* is explained by inhibition of angiogenesis [5]. Studies support this by stating the comparability with antineoplastic drug paclitaxel, followed with the absence of any signs of acute toxicity [32]. The second abundant biologically active compound in the examined extract exhibiting antiproliferative action on numerous human cancer cell lines was hypericin. The proposed mechanism of hypericin antiproliferative effects is also apoptosis [5]. Beyond hyperforin and hypericin, it was also reported that flavonoids, such as quercetin, inhibit cancer cells growth [36]. However, cytotoxicity of* H. perforatum* extracts in our research presents the result of joined action of major constituents, hyperforin and hypericin, as well as other secondary metabolites present in the investigated extract.

**Figure 3:**
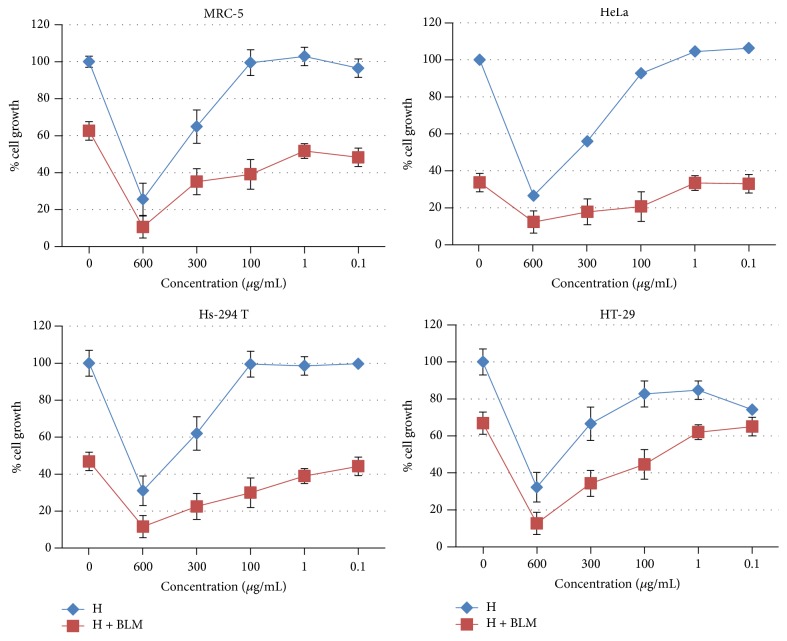
Cytotoxicity of HP and combination of HP and BLM (100 *μ*g/mL) during 48 h treatment. The results are expressed as mean ± SD performed in octuplicate.

On the other hand, significant antitumor activity was recorded during the treatment of selected cell cultures by series of concentrations of combination of HP and BLM (100 *μ*g/mL) (Table 2, Figure 3). Selective cytotoxicity of this combined treatment was considerably high on cervix cancer cells (HeLa), as well as on melanoma cancer cells (Hs-294T), while it was low on colon cancer cells (HT-29). By comparing the EC_50_ values of different tumor cell lines it can be noticed that HeLa are the most susceptible to the treatment by combination of the HP and BLM, followed by Hs-294T, while HT-29 are the most resistant. It is known that products of natural origin, in combination with conventional medicines, can affect the efficacy of the applied therapy by interfering in signaling cascades of transport proteins and enzymes and up- or downregulating the transport and metabolism [11]. Bleomycin is radiomimetic which induces DNA damage as ionizing radiation (IR). As a result of its action, formed DNA strain breaks lead toward the production of DNA adducts and excess ROS which induce oxidative stress, mitochondrial leakage, and apoptosis [37]. It is important to highlight that BLM belongs to the group of drugs associated with radiation sensitivity [38]. Previous reports suggest that hypericin increases the sensitivity of cancer cells to ionizing radiation which puts it in a group of potential new agents in combination with radiation therapy of malignant gliomas [39]. Furthermore, significant level of selectivity toward cervix carcinoma and melanoma cell lines against healthy cells was achieved, described by NT/T [40]. Altogether, it is obvious that the cellular events leading to the increment of cytostatic effect as a result of cotreatment by HP and BLM are complex and should be investigated further.

### 3.5. Genotoxicity

**Figure 4:**
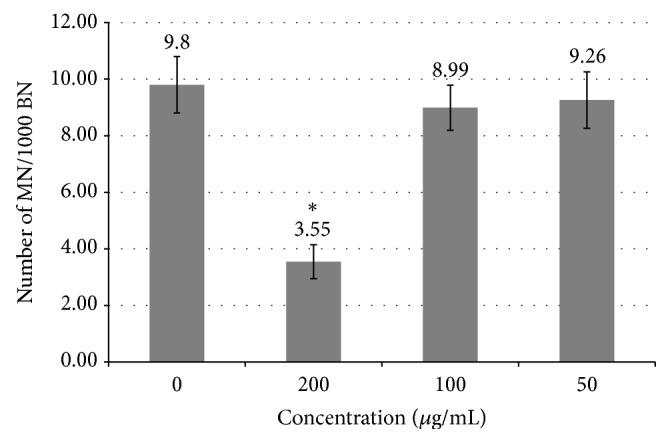
Incidence of micronuclei by HP during 24 h treatment. MN: micronuclei; BN: binucleated cells. The results are shown as mean ± SD performed in triplicate. Concentrations were expressed in *μ*g/mL. ^*∗*^Statistically significantly different from the control (Fisher LSD test; *p* < 0.05).

Genotoxicity assays enable the assessment of mutagenic and cancerogenic potential of different extracts of medicinal plants and their protective effects, as well as mechanisms of their biological activities. These assays are used to identify the extracts with the ability to react with nucleic acids in subcytotoxic concentrations. If the substance damages the genome, it is manifested by the increment of incidence of micronuclei. Genotoxicity of the HP was tested by micronucleus assay on lymphocytes of peripheral blood during the 24 h treatment. The results of micronuclei incidence are shown in Figure 4. During the treatment HP induced decreasing of micronuclei incidence which was lower compared to the control value. Statistically significant decrease of micronuclei (MN: 3.55 versus 9.80; *p* = 0.008) was observed at concentration of 200 *μ*g/mL. Ramos et al. [41] reported that* H. perforatum* exhibits antigenotoxic effects on HT-29 cells which corresponds to the result of our study. Namely, it was stated that the extracts of* H. perforatum* have protective effect against oxidative DNA damage followed by increasing of the repair of alkylating DNA damage by base excision repair pathway. These protective properties were ascribed to the high amount of phenolic compounds. In another* in vitro* study of St. John's wort, extracts rich in flavonoids exhibited strong antioxidant activity and radical scavenging characteristics [42]. Considering that the current study confirms notable antioxidant potential of HP, decreasing of the micronuclei incidence is the expected result.

## 4. Conclusions

The results of the conducted study concerning the chemical profiling of* H. perforatum* subsp.* perforatum* during flowering and fruitification suggest that the maximum accumulation of hypericin and hyperforin is present in the transition period between floral budding and full flowering stage.* Hyperici herba* collected at aforementioned time interval will also contain significant amounts of naringenin, rutin, quercetin, amentoflavone, chlorogenic, caffeic, and* p*-hydroxybenzoic acid. The evaluation of biological potential of the prepared water-alcoholic extract from the plant material collected at the stage of full flowering suggests notable antioxidant potential, comparable to synthetic antioxidants. Also, the potential of the extract to inhibit biologically important enzymes cannot be neglected, especially in the case of *α*-glucosidase which opens a place for further evaluation of St. John's wort as antihyperglycemic agent. The combination of HP at concentrations higher than 1 *μ*g/mL and bleomycin (100 *μ*g/mL) leads to significant increment of antiproliferative effects on cancer cell lines, while the examined extract does not show signs of genotoxicity at subcytotoxic concentrations (<200 *μ*g/mL). However, during evaluation of biological potential of different plant extracts, the fact that the evaluated subject (in this case the St' John's wort extract) is a mixture of compounds with different characteristics exhibiting various biological effects must be always taken into the account.

## Figures and Tables

**Table 1:** Detailed chemical characterization of *H. perforatum* subsp. *perforatum* water-alcoholic extracts by HPLC - DAD.

Date of sampling/phenological stage	Compound		Hpf	Hpc	Ap	NA	Ec	R	Qe	Am	FA	GA	CHA	CA	PHB	% d. e.
	Sample		mg/g d. h.													
20.04.2014. BF^*∗∗*^	1	Mean value	0.4113	0.1277	0.0048	0.0378	n. d.	0.0871	0.0654	0.0083	n. d.	0.1735	0.0742	0.0613	0.0716	17.66
		SD	0.0001	0.0026	0.0001	0.0016	n. d.	0.0033	0.0022	0.0001	n. d.	0.0011	0.0013	0.0025	0.0028	
27.04.2014. BF	2	Mean value	0.4565	0.1359	0.0060	0.0475	n. d.	0.1235	0.0721	0.0138	n. d.	0.1321	0.0733	0.0600	0.0689	23.57
		SD	0.0052	0.0057	0.0002	0.0023	n. d.	0.0059	0.0022	0.0001	n. d.	0.0065	0.0028	0.0009	0.0034	
04.05.2014. BF	3	Mean value	0.4325	0.1498	0.0057	0.0498	n. d.	0.1300	0.0975	0.0140	n. d.	0.0249	0.0622	0.0573	0.0753	22.27
		SD	0.0003	0.0018	0.0000	0.0006	n. d.	0.0041	0.0021	0.0001	n. d.	0.0006	0.0015	0.0012	0.0031	
11.05.2014. BF	4	Mean value	0.4142	0.1389	0.0060	0.0496	n. d.	0.1363	0.0942	0.0117	n. d.	0.0251	0.0635	0.0552	0.0716	23.17
		SD	0.0132	0.0018	0.0003	0.0018	n. d.	0.0031	0.0040	0.0002	n. d.	0.0004	0.0009	0.0013	0.0031	
18.05.2014. BF	5	Mean value	0.4569	0.1689	0.0095	0.0513	n. d.	0.1506	0.1155	0.0144	n. d.	0.0275	0.0766	0.0548	0.0818	23.49
		SD	0.0061	0.0047	0.0004	0.0006	n. d.	0.0048	0.0024	0.0005	n. d.	0.0003	0.0019	0.0023	0.0020	
25.05.2014. BF	6	Mean value	0.4020	0.1918	0.0116	0.0523	n. d.	0.1050	0.1024	0.0149	n. d.	0.0146	0.0636	0.0533	0.0750	23.23
		SD	0.0034	0.0002	0.0003	0.0026	n. d.	0.0046	0.0035	0.0004	n. d.	0.0000	0.0025	0.0025	0.0020	
01.06.2014. FB^*∗∗*^	7	Mean value	0.4305	0.2107	0.0090	0.0550	n. d.	0.0904	0.1237	0.0130	n. d.	0.0165	0.1283	0.0654	0.0807	23.56
		SD	0.0161	0.0013	0.0004	0.0004	n. d.	0.0039	0.0014	0.0005	n. d.	0.0002	0.0012	0.0017	0.0004	
04.06.2014. FB	8	Mean value	0.6401	0.3370	0.0057	0.0742	n. d.	0.0879	0.1359	0.0248	n. d.	0.0147	0.1255	0.0674	0.0774	23.87
		SD	0.0205	0.0020	0.0002	0.0018	n. d.	0.0028	0.0031	0.0010	n. d.	0.0003	0.0034	0.0032	0.0023	
08.06.2014. FB	9	Mean value	0.8934	0.4987	0.0021	0.1259	n. d.	0.0836	0.1346	0.0456	n. d.	0.0266	0.1292	0.0746	0.0841	20.47
		SD	0.0443	0.0128	0.0001	0.0055	n. d.	0.0005	0.0038	0.0001	n. d.	0.0001	0.0034	0.0034	0.0005	
11.06.2014. F^*∗∗*^	10	Mean value	0.9237	0.5913	n. d.^*∗*^	0.1206	n. d.	0.0800	0.1220	0.0417	0.0351	0.0275	0.1274	0.0707	0.0653	21.80
		SD	0.0131	0.0012	n. d.	0.0003	n. d.	0.0002	0.0019	0.0020	0.0005	0.0001	0.0048	0.0029	0.0010	
15.06.2014. F	11	Mean value	0.9195	0.6632	n. d.	0.1253	n. d.	0.0799	0.1197	0.0402	0.0350	0.0193	0.0642	0.0509	0.0580	18.66
		SD	0.0355	0.0257	n. d.	0.0041	n. d.	0.0027	0.0045	0.0009	0.0008	0.0003	0.0030	0.0013	0.0022	
18.06.2014. F	12	Mean value	0.8666	0.6987	n. d.	0.1159	n. d.	0.0805	0.1224	0.0381	0.0369	0.0077	0.0636	0.0483	0.0515	16.67
		SD	0.0210	0.0026	n. d.	0.0029	n. d.	0.0016	0.0029	0.0007	0.0008	0.0002	0.0025	0.0017	0.0013	
22.06.2014. F	13	Mean value	0.7541	0.7811	n. d.	0.1100	n. d.	0.0749	0.1104	0.0334	0.0369	n. d.	0.0615	0.0508	0.0533	14.34
		SD	0.0114	0.0171	n. d.	0.0045	n. d.	0.0004	0.0003	0.0002	0.0002	n. d.	0.0024	0.0004	0.0006	
25.06.2014. F	14	Mean value	0.7005	0.7857	n. d.	0.1086	n. d.	0.0737	0.0985	0.0326	0.0401	n. d.	0.0692	0.0450	0.0388	14.21
		SD	0.0191	0.0148	n. d.	0.0046	n. d.	0.0023	0.0029	0.0004	0.0017	n. d.	0.0006	0.0006	0.0008	
03.07.2014. AF^*∗∗*^	15	Mean value	0.6379	0.8852	n. d.	0.1076	0.0469	0.0621	0.0887	0.0333	0.0369	n. d.	0.0736	0.0412	0.0390	19.29
		SD	0.0002	0.0214	n. d.	0.0031	0.0020	0.0005	0.0032	0.0010	0.0010	n. d.	0.0004	0.0011	0.0014	
06.07.2014. AF	16	Mean value	0.5489	0.6769	n. d.	0.0840	0.0599	0.0609	0.0799	0.0292	0.0415	n. d.	0.0633	0.0430	0.0337	18.68
		SD	0.0168	0.0332	n. d.	0.0001	0.0026	0.0019	0.0006	0.0001	0.0005	n. d.	0.0013	0.0016	0.0012	
13.07.2014. AF	17	Mean value	0.4740	0.5560	n. d.	0.0815	0.0587	0.0615	0.0712	0.0289	0.0351	n. d.	0.0580	0.0357	0.0339	13.54
		SD	0.0192	0.0082	n. d.	0.0039	0.0011	0.0008	0.0009	0.0003	0.0015	n. d.	0.0025	0.0012	0.0009	
20.07.2014. AF	18	Mean value	0.5073	0.5450	n. d.	0.0834	0.0570	0.0587	0.0787	0.0287	0.0413	n. d.	0.0637	0.0331	0.0344	14.10
		SD	0.0082	0.0072	n. d.	0.0028	0.0011	0.0013	0.0031	0.0011	0.0000	n. d.	0.0021	0.0003	0.0008	
27.07.2014. AF	19	Mean value	0.6582	0.5269	n. d.	0.0862	0.0490	0.0573	0.0778	0.0302	0.0369	n. d.	0.0568	0.0343	0.0344	10.34
		SD	0.0019	0.0144	n. d.	0.0016	0.0024	0.0028	0.0014	0.0010	0.0016	n. d.	0.0004	0.0012	0.0005	
30.07.2014. AF	20	Mean value	0.6898	0.4987	n. d.	0.0854	0.0315	0.0615	0.0947	0.0308	0.0386	n. d.	0.0543	0.0321	0.0337	10.76
		SD	0.0013	0.0182	n. d.	0.0041	0.0006	0.0021	0.0001	0.0012	0.0015	n. d.	0.0013	0.0006	0.0012	
03.08.2014. AF	21	Mean value	0.7568	0.5174	n. d.	0.0862	0.0315	0.0611	0.0990	0.0307	0.0369	n. d.	0.0686	0.0369	0.0428	12.2
		SD	0.0127	0.0165	n. d.	0.0012	0.0015	0.0005	0.0011	0.0013	0.0001	n. d.	0.0010	0.0008	0.0000	
06.08.2014. AF	22	Mean value	0.8121	0.4786	n. d.	0.0853	0.0326	0.0561	0.1015	0.0291	0.0369	n. d.	0.0590	0.0345	0.0450	12.33
		SD	0.0020	0.0054	n. d.	0.0023	0.0004	0.0012	0.0050	0.0012	0.0002	n. d.	0.0029	0.0013	0.0003	

^*∗*^n.  d.: not detected; ^*∗∗*^BF: before flowering; FB: floral budding; F: flowering; AF: after flowering.

**Table:** (a) Antioxidant potential

		Radical scavenging capacity (RSC)			LP	FRAP
		DPPH^•^	OH^•^	NO^•^		
RSC_50_ (*µ*g/mL)	HP	3.68 ± 0.12	66.99 ± 3.15	139.79 ± 5.98	471.72 ± 11.25	212.26 ± 3.59
	BHT	/	0.03 ± 0.01	/	7.32 ± 0.56	/
	Ascorbic acid	/	2.05 ± 0.11	/	/	/
	QDH	0.97 ± 0.02	/	/	/	/
	PG	0.69 ± 0.04	9.09 ± 0.59	9.27 ± 0.39	/	/

**Table:** (b) Inhibition of biologically important enzymes

		MAO-A	MAO-B	AChE		*α*-Amylase		*α*-Glucosidase	
		% of inhibition							
Conc. (*µ*g/mL)		4.09	73.53	475	800	400	800	8.32	16.64

	HP	20.46 ± 1.59	86.69 ± 3.47	17.22 ± 1.36	42.31 ± 4.18	51.10 ± 3.29	90.78 ± 2.97	41.33 ± 2.19	71.01 ± 3.67

IC_50_ (*µ*g/mL)	Moclobemide	0.64 ± 0.04	/	/		/		/	
	Selegiline	/	0.23 ± 0.02	/		/		/	
	Galantamine	/	/	8.56 ± 0.77		/		/	
	Acarbose	/	/	/		4.93 ± 0.33		42.87 ± 3.43	

**Table:** (c) Cytotoxic potential

	MRC-5	HeLa	Hs-294 T	HT-29	HeLa	Hs-294 T	HT-29
	EC_50_ (*µ*g/mL)				NT/T		
HP	480.62 ± 20.15	340.94 ± 12.74	468.94 ± 14.47	342.46 ± 11.67	1.40	1.02	1.40
HP + BLM	2.76 ± 0.21	0.02 ± 0.00	0.05 ± 0.01	4.21 ± 0.14	108.04	53.60	0.65
BLM	80.3 ± 4.57	77.2 ± 5.14	45.18 ± 2.34	165.31 ± 5.89	1.04	1.77	0.48

*Note.* NT/T: nontumor/tumor cells EC_50_ ratio.
